# Benthic communities on recent and historical volcanic lava flows of an oceanic island

**DOI:** 10.1371/journal.pone.0357447

**Published:** 2026-09-23

**Authors:** Aitor Ugena, Sara González-Delgado, Iván Cano, Marina Aliende-Hernández, Beatriz Alfonso, José Carlos Hernández

**Affiliations:** 1 Marine Community Ecology and Conservation, Dpto. Biología Animal, Edafología y Geología, Universidad de La Laguna, Tenerife, Canary Islands, Spain; 2 Dpto. Biologia Evolutiva, Ecologia i Ciencies Ambientals, Facultat de Biologia and Institut de Recerca de la Biodiversitat (IRBio), Universitat de Barcelona, Barcelona, Spain; 3 Marine Community Ecology and Conservation, Dpto. Botánica, Ecología y Fisiología Vegetal, Universidad de La Laguna, Tenerife, Canary Islands, Spain; IEAPM: Instituto de Estudos do Mar Almirante Paulo Moreira, BRAZIL

## Abstract

Understanding how benthic communities develop on newly formed marine substrates is essential for explaining ecological succession after large natural disturbances. However, different benthic components may respond at different temporal scales. Here, we examined successional patterns in selected components of shallow benthic communities across three submarine lava flows of contrasting ages on La Palma Island (Canary Islands, Spain): less than 1 year, 73 years, and 473 years old. Specifically, we focused on macroalgal assemblages, early post-settlement microinvertebrates collected using artificial collectors, and established rock-associated microinvertebrate communities. To do so, we combined underwater photoquadrats, settlement collectors, and direct sampling of rock-associated microinvertebrates to compare successional patterns among these three benthic components. Species richness and community composition differed among substrate ages, but the strength and shape of the response varied among biological components. Macroalgal richness was lowest on the youngest flow and showed similar values between the intermediate and oldest substrates, suggesting relatively early compositional stabilization. In contrast, established rock-associated microinvertebrates showed maximum richness and taxonomic distinctiveness on the 73-year-old lava flow, followed by greater dominance of specialized and predatory taxa on the oldest flow. Early post-settlement assemblages showed intermediate patterns, linking initial colonization to the development of mature benthic communities. The ratio between newly settled and successfully established taxa declined with substrate age, suggesting an increasing role of post-settlement filtering as communities matured. Overall, our results show that successional patterns differed among benthic components, with macroalgal assemblages showing early compositional stabilization, whereas established rock-associated microinvertebrates exhibited a clearer richness peak at the intermediate successional stage. These patterns highlight the combined role of substrate age, habitat structure, and post-settlement processes in shaping selected components of shallow benthic assemblages on volcanic coasts.

## 1. Introduction

Volcanic islands represent dynamic environments where geological and biological processes interact across both short and long temporal scales [1]. Volcanic eruptions, while often perceived as purely destructive, dramatically reshape coastal and marine landscapes by depositing fresh lava flows that alter existing habitats and create new substrates. These events provide unique natural laboratories for studying primary ecological succession, as they effectively “reset” local ecosystems, eliminating established biological communities in some areas and exposing entirely new, uncolonized surfaces [2,3].

The Canary Islands constitute a volcanic archipelago that has formed progressively over the past 60 million years [4]. Throughout this period, at least 17 eruptions have been documented across different islands, continuously reshaping both terrestrial and submarine landscapes [5]. Volcanic activity remains ongoing, as exemplified by the 2011 Tagoro submarine eruption near El Hierro Island [6] and the 2021 Tajogaite eruption in La Palma Island [7]. The Tagoro event, in particular, deposited large volumes of new material that buried preexisting benthic communities, initiating a new phase of ecological succession characterized by the colonization of pioneering organisms, including macroalgae, annelids, crustaceans, cnidarians, and mollusks [6,8].

La Palma Island has undergone eight documented volcanic eruptions, beginning with the Tacande eruption in 1430 [7,9]. The most recent event, the Tajogaite eruption (September–December 2021), profoundly altered both terrestrial and marine environments. As lava entered the sea along the southern coast of Tazacorte municipality, it gave rise to two new lava deltas: a smaller one extending ~0.5 km from the original shoreline, and a larger southern delta spanning ~1.2 km, partially overlapping deposits from the 1949 San Juan eruption [10]. These new formations buried preexisting habitats and extended the coastline seaward, altering local hydrodynamics and sediment regimes. Prior to the eruption, the seabed comprised shallow rocky substrates transitioning into sandy bottoms [11]. At the delta fronts, lava‑seawater interactions produced transient perturbations in carbonate chemistry, including acidification plumes and altered alkalinity profiles [12]. Furthermore, the newly formed basaltic substrates constitute highly heterogeneous and dynamic bottoms, influencing colonization pathways and taxonomic turnover in benthic assemblages [13]. Recent work on the Tajogaite lava flows has provided important insights into the early primary succession of marine communities in this volcanic system, including broad patterns of colonization by fishes, macroinvertebrates, and alga [13]. Our study provides a complementary perspective by focusing on macroalgal assemblages, early post-settlement microinvertebrates, and established rock-associated microinvertebrate communities across lava flows of contrasting ages.

These conditions on La Palma Island offer a unique opportunity to examine the formation and development of selected components of shallow marine benthic communities on newly created volcanic substrates. Moreover, the island’s recurrent volcanic activity has produced seafloor habitats of markedly different ages coexisting within a relatively small geographic area. In addition to the recent lava flows generated during the 2021 Tajogaite eruption, older submarine substrates shaped by the San Juan eruption approximately 73 years ago and the Jedey eruption around 473 years ago are also present. This chronological gradient of substrate ages constitutes a unique natural ecological timeline, enabling direct comparisons of the assembly of macroalgal and associated microinvertebrate assemblages across different successional stages from the initial colonization of bare rock to the development of mature and stabilized assemblages.

Ecological succession is a dynamic process characterized by increasing biomass, biodiversity, and ecological complexity, shaped by the interplay of biotic interactions and abiotic factors [14–16]. In marine environments, colonization tends to occur more rapidly than on land, facilitated by ocean currents and the high dispersal potential of many marine organisms [17,18]. This promotes the swift arrival of pioneering species and the subsequent formation of nascent communities, as observed in post-eruption environments such as Kasatochi Island and Vailulu'u seamount [3,19]. The first colonizers often include microbial mats, microalgae and opportunistic macroalgae, which are soon followed by copepods, amphipods, and early crustaceans such as brachyuran and galatheid crabs [20,21]. These species are typically characterized by low competitive ability but high demographic plasticity, exhibiting traits such as physiological tolerance to extreme conditions (e.g., temperature and salinity), broad dietary flexibility, rapid reproduction, and strong dispersal capacity [15,22,23]. By modifying the habitat, these early colonizers facilitate the arrival and establishment of additional species by increasing surface complexity, food availability, and refuge availability. These habitat-mediated effects are particularly relevant for mobile epifauna, whereas sessile or tube-building invertebrates may depend more strongly on substrate stability and the progressive development of consolidated microhabitats.

In the intermediate successional stage, diversity often peaks due to the coexistence of pioneer species and later-arriving taxa [24]. Conditions such as increased organic matter, substrate stabilization, and moderate hydrodynamics enable the development of larger, more structurally complex macroalgae. In subtropical latitudes taxa such as *Dictyota humifusa* and *Sargassum* spp. may exemplify this stage when present, although some macroalgae, including canopy-forming or opportunistic taxa, can show seasonal fluctuations in abundance and cover. Therefore, the macroalgal assemblages recorded in this study should be interpreted within the summer sampling context [25]. As succession advances, the community structure shifts from generalist, fast-reproducing taxa to more specialized or competitively dominant organisms [14,16]. This transition reflects increasing ecological complexity and niche partitioning, where late-successional species exhibit slower growth, narrower habitat preferences, and superior abilities to compete for space and resources. Ultimately, the community may reach a relatively stable “climax” state, often characterized by lower diversity but higher specialization and structural integrity [16]. Crucially, these transitions are shaped not only by initial settlement but also by post-settlement processes, including what is often referred to as post-settlement filtering. This concept describes the selective establishment of species after arrival, mediated by habitat suitability, biotic interactions, predation, competition, and prevailing environmental conditions [26]. In this context, species considered “competitive” are those capable of thriving in mature, stable environments due to traits such as superior resource acquisition, effective space occupation, and ecological specialization, which distinguish them from the generalist strategies typical of early colonizers [14,15]

By examining a unique timeline, of lava substrates of different ages on La Palma Island (<1 year, 73 years, and 473 years), this study reconstructs successional trajectories across a temporal gradient following volcanic disturbance. We combined *in situ* surveys of macroalgal and benthic microinvertebrate communities (i.e., defined here as invertebrates between 0.06 mm and 10 mm associated with algal surfaces, rocky substrates, or biofilm layers) with settlement traps designed to record larval arrivals and early post-settlement stages. This dual approach allowed us to document both the composition of established communities and the initial colonization dynamics. Our objective was to determine how macroalgal assemblages, early post-settlement microinvertebrates, and established rock-associated microinvertebrate communities vary across lava flows of contrasting ages, and to explore whether the observed patterns are consistent with progressive successional development and increasing post-settlement filtering. We hypothesized that richness and composition would differ among lava flows of contrasting ages, with the intermediate-aged substrate potentially supporting higher richness in some components due to the coexistence of early colonizers and later-arriving taxa. We also expected that differences between early post-settlement assemblages and established rock-associated communities would provide indirect evidence of stronger post-settlement filtering in older, more mature substrates. Additionally, we expected that post-settlement filtering processes would intensify with community maturity, favoring more specialized and competitively dominant taxa in the oldest lava flow.

## 2. Materials and methods

### 2.1 Study area

The sampling was conducted at three sites along the coast of La Palma (Canary Islands, Spain), beach representing volcanic rocky substrates of different ages (<1 year, 73 years, and 473 years). To minimize environmental variability associated with the island’s exposure to prevailing trade winds and dominant currents, all sites were selected along the southwest-facing coastline of La Palma. In addition, the three study sites are situated within a coastal stretch of approximately 10 km, ensuring close spatial proximity and broadly comparable coastal and benthic settings. This spatial configuration likely reduces large-scale differences in hydrodynamic regime, wave exposure, substrate heterogeneity, extent of rocky platforms, vertical relief, and sedimentation patterns [27]. Although we acknowledge that direct measurements of these environmental variables would have further strengthened the study, such measurements were beyond the possibilities of the present work.

**Fig 1:**
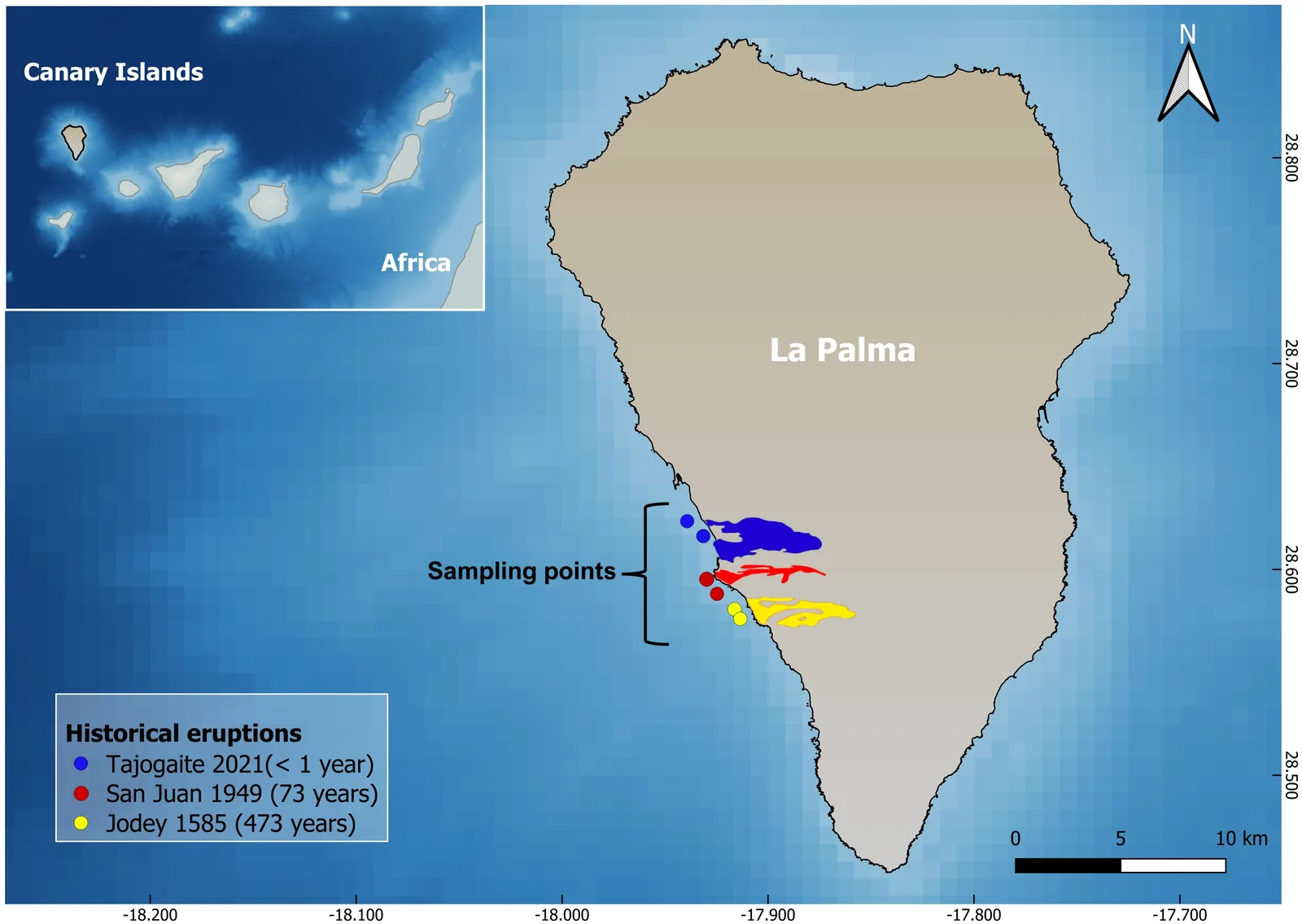
Map of La Palma Island showing sampling points at the three studied lava flows. The bathymetric base map was generated by the authors using the marmap package in R, with public domain data from the NOAA (National Oceanic and Atmospheric Administration). Lava flow geometries for Tajogaite (2021), San Juan (1949), and Jodey (1585) were created by the authors based on open data from the Instituto Geográfico Nacional (IGN).

This orientation is also with the best representation of lava flows of different ages that reached the sea. The oldest sampled area corresponds to the Jedey lava flow, Puerto Naos; 28.584996° N, 17.910301° W, produced by a Strombolian eruption that began in May 1585 and lasted several weeks [28]. The intermediate-aged substrate is the San Juan lava flow, La Bombilla; 28.592422° N, 17.919193° W, resulting from a fissural eruption in June 1949 that persisted for 42 days [29]. The most recent substrate stems from the 2021 Tajogaite eruption, Playa del Arco; 28.609141° N, 17.923526° W, also fissural in nature, which erupted in September 2021 and continued for two months and 24 days. This last event reached the sea, creating two new land formations measuring approximately 5.05 ha (northern area) and 43.46 ha (southern area) (Fig 1).

### 2.2 Sampling

Sampling was conducted by a team of three divers in August 2022 at each selected lava flow at two sites, at 10 meters depth. The sampling design encompassed three complementary approaches: photographic surveys of macroalgae, artificial settlement collectors for early colonizers, and direct sampling of rocky substrates to characterize benthic microinvertebrate communities. Details of each method are provided below.

In this study, the term “microinvertebrates” refers to individuals between 0.06 mm and 10 mm recorded in the samples, including larvae, juveniles, early settlers, and small cryptic stages associated with algal surfaces, rocky substrates, or biofilm layers. This definition refers to the size or life stage of the individuals collected, rather than to the maximum adult size of the species. Thus, taxa whose adults may exceed 10 mm were included only when the individuals collected corresponded to larvae, juveniles, early settlers, or small cryptic stages within the operational size range considered.

For each lava flow, sampling was conducted at two independent sites separated 500 meters away. This sampling design allows us to replicate volcanic substrates with the same age at the same island. It is important to highlight here that this set of lava flows is unique, and no other lava flows of same age can be found at least for 0 years and 73 years old.

**Fig 2:**
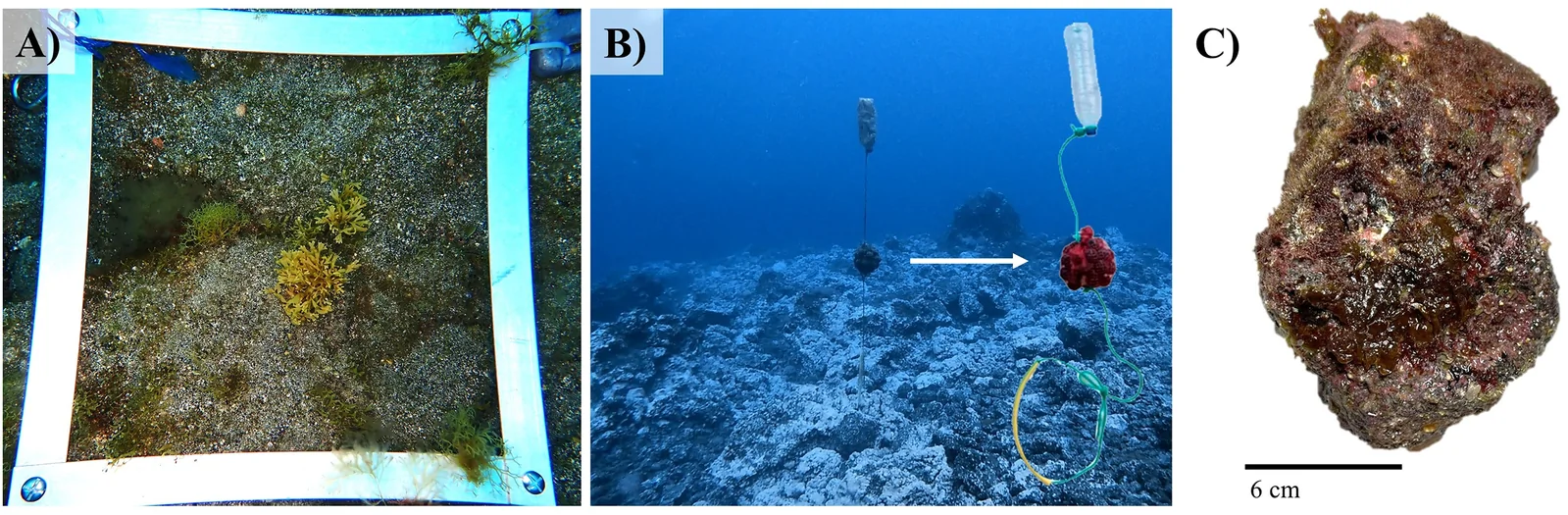
Sampling methodologies used in the study. **A:** Macroalgae cover photoquadrats; **B:** Early settlers’ collectors; C: Collected rock sample. All photographs were taken by the authors during fieldwork.

To assess the algal community, standardized 25 × 25 cm photoquadrats (Fig 2A) were randomly deployed across all three volcanic substrates. This design yielded 40 photoquadrats per lava flow (3 lava flows × 2 sites × 20 replicates) and 120 photoquadrats in total across the three lava flows. These images provided a non-invasive record of algal composition and coverage, using an OLYMPUS TG6 underwater camera. All the images were later analyzed to identify macroalgal taxa and quantify their presence and percent cover, allowing the characterization of macroalgal assemblage composition.

To investigate early microinvertebrate settlement, artificial collectors were deployed on each lava flow (Fig 2B). Each collector consisted of a hollow plastic sphere with a diameter of 7.5 cm, surrounded by 30 polypropylene biofilter balls, each 3.9 cm in diameter and with an approximate surface area of 0.04 m². The biofilter balls provided standardized artificial substrate and interstitial spaces for larval settlement, juvenile recruitment, and colonization by small mobile invertebrates. To prevent the loss of biofilter balls, each device was enclosed in a nylon net and anchored to the seabed with a plastic rope. Specifically, three collectors were installed at each of two spatially separated sites per lava flow, at a depth of 10 m, resulting in six collectors per lava flow and 18 collectors in total. Collectors were left in situ for 30 days to allow settlement. After retrieval, each collector was carefully rinsed in the laboratory, and all retained material was preserved in 95% ethanol at 4 °C until further processing [30]. Samples were subsequently examined under a stereomicroscope, and larvae, juveniles, and early settlers were sorted and identified to the lowest possible taxonomic level. As is common in field-based deployment experiments, some collectors could not be recovered after the 30-day exposure period due to environmental conditions, leading to minor differences in sample size among lava flows.

To characterize the benthic microinvertebrate community associated with consolidated substrates, rocky samples were collected from the same sites and depth as the settlement collectors. At each site, three rocks measuring approximately 15–20 cm in diameter were carefully collected by hand from the substrate at 10 m depth and placed individually into sealed plastic bags to minimize the loss of associated organisms, yielding a total of 18 rock samples across all lava flows (Fig 2C). In the laboratory, each rock was photographed from all faces, including the surface previously in contact with the substrate. These photographs were used to estimate the sampled surface area of each rock, allowing data to be standardized by sample area, since rocks of similar diameter may still differ in total surface area. The images also provided a permanent visual record of cryptic microhabitats on the underside of stones. All images were taken using an OLYMPUS TG-6 camera. Rocks were then thoroughly examined, and all sessile and slow-moving microinvertebrates were removed, counted, and identified following the taxonomic procedures described in section 2.3. This approach provided detailed information on community composition and abundance that is not readily captured through *in situ* visual or photographic surveys alone.

### 2.3 Laboratory work

Laboratory processing was conducted separately for macroalgal photoquadrats, artificial settlement collectors, and rock-associated microinvertebrates. Macroalgal assemblages were analyzed from photoquadrats using the open-source image analysis software ImageJ v1.53v [31], which allowed the identification of taxa and the estimation of their percent cover. Whenever possible, macroalgae were identified to the lowest feasible taxonomic level based on morphological characteristics visible in the photographs, using standard identification guides for the Canary Islands [32]. The taxonomic list of macroalgae species collected in the lava-flows is provided in Supplementary S1 Table.

Due to the limitations inherent to photographic identification, crustose coralline algae could not be reliably distinguished at species level and were therefore grouped as crustose coralline algae (*Corallina* spp), and the same with epiphytic algae, grouped in epiphytes spp. This methodological constraint is acknowledged, as species-level resolution of coralline algae would require destructive sampling or microscopic analyses beyond the scope of the present study.

For the analysis of early post-settlement assemblages, organisms were carefully extracted from the collectors upon retrieval, counted, photographed, and identified to the lowest possible taxonomic level. For rock-associated microinvertebrates, each collected rock was placed in a tray and all organisms were carefully removed using fine forceps, counted, and identified. All early post-settlement and rock-associated microinvertebrates were examined under a stereomicroscope (Leica EZ4) using diagnostic external morphological characters and specialized taxonomic literature for the main groups recorded. Decapod crustaceans were identified following [33], echinoderms following [34], molluscs following [35], and cyclostome bryozoans following [36]. Sipunculids were identified using specialized literature for the group [37]. Polychaetes were identified based on diagnostic external characters, including body segmentation, chaetal arrangement, tube morphology, branchiae, and opercular or radiolar structures when present; species-level identifications were supported and confirmed by Dr. Jorge Núñez, a specialist in polychaete taxonomy. REDPROMAR (https://redpromar.org/species, accessed in 2024) was used as a complementary regional reference, and WoRMS (https://www.marinespecies.org/index.php, accessed in 2024) was used to verify current taxonomic nomenclature and accepted species names.

When diagnostic characters were insufficient for confident species-level identification, specimens were assigned to genus, family, or morphospecies level and treated consistently in the richness and community composition analyses. In the few cases where taxonomic resolution could only be achieved at genus level, each genus-level category represented a single distinguishable taxon, and ambiguous pooled categories were avoided. The complete list of identified microinvertebrates taxa in the lava-flows’ is provided in Supplementary S2 Table.

### 2.4 Statistical analysis

Statistical analyses were conducted to examine patterns of biodiversity and community structure across lava flows of different ages (< 1 year, 73 years, and 473 years) for three selected components of shallow benthic assemblages: (1) macroalgae, (2) early post-settlement microinvertebrates collected using artificial collectors, and (3) established rock-associated benthic microinvertebrates.

Species richness was analysed using a univariate modelling framework based on generalized linear models (GLMs). Richness, defined as the number of species per sample, was modelled as a function of lava flow age using count-based distributions with a log-link function. Preliminary diagnostics indicated deviations from standard Poisson assumptions in some datasets; therefore, Conway–Maxwell–Poisson (COM-Poisson) error structures were implemented when required to appropriately account for dispersion patterns in the data.

For each of the three analysed components, namely macroalgal assemblages, early post-settlement microinvertebrates, and established rock-associated microinvertebrate communities, lava flow age was included as a fixed factor with three levels (< 1, 73, and 473 years). Model adequacy was assessed through residual diagnostics (Supplementary S3 Table*)*, including simulation-based methods. Global effects of lava flow age on species richness were evaluated using likelihood ratio tests. When significant effects were detected, pairwise comparisons among lava flows were conducted using estimated marginal means with Tukey-adjusted p-values. Model predictions and associated 95% confidence intervals were used to visualize richness patterns across the successional gradient.

To evaluate differences in community composition and species abundance among lava flows, multivariate analyses were performed. Abundance data were square-root transformed to reduce the influence of dominant taxa, and Bray–Curtis similarity matrices were constructed. Differences in community composition among lava flows were tested using permutational multivariate analysis of variance (PERMANOVA). Significant results (p < 0.05) were followed by pairwise post-hoc comparisons. Non-metric multidimensional scaling (nMDS) ordinations were used to visualize similarity patterns among samples. Additionally, SIMPER (Similarity Percentage) analyses were conducted to identify the species contributing most to observed compositional differences between lava flows.

Before testing the effect of lava flow age, preliminary analyses were conducted to assess potential differences between the two sampling sites within each lava flow for both species richness and community composition. No significant site-level differences were detected within lava flows (all P > 0.05; S4 Table). Given the limited number of sites per lava flow and the absence of detectable within-flow site effects, samples were analysed as within-flow replicates, and subsequent analyses focused on lava flow age as the main explanatory factor.

All multivariate analyses were performed using PRIMER v7 with the PERMANOVA+ add-on [38].

### 2.5 Permits and ethical statement

Field sampling was conducted under the corresponding authorizations issued by the Viceconsejería de Medio Ambiente del Gobierno de Canarias and in accordance with the Special Civil Protection and Emergency Response Plan for Volcanic Risk in the Canary Islands (PEVOLCA). Sampling was carried out in public shallow subtidal rocky habitats along the coast of La Palma, and no access to privately owned land was required. No protected species were intentionally collected or sampled during this study. The study did not involve human participants, vertebrate animals, cephalopods, vertebrate embryos, or human tissue. Therefore, no animal husbandry, experimental maintenance, anesthesia, euthanasia, or animal care/welfare procedures were involved.

## 3. Results

### 3.1 Macroalgae community

Across the three lava flows, 12 macroalgal taxa were recorded in total, with clear differences in cover, composition, and assemblage structure among substrate ages. The youngest lava flow (<1 year) supported 5 taxa and was characterized by very low macroalgal cover and a high proportion of uncolonized substrate. This initial assemblage was mainly composed of perennial crustose Corallinales together with seasonal opportunistic taxa such as *Cottoniella filamentosa* and *Pseudotetraspora marina*. The intermediate-aged lava flow (73 years) supported 11 taxa and showed a marked increase in macroalgal cover, being strongly dominated by the seasonal and opportunistic filamentous red alga *Lophocladia trichoclados*. The oldest lava flow (473 years) supported 10 taxa and exhibited a more structurally complex assemblage, characterized by the co-occurrence of *L. trichoclados* with perennial corticated and leathery macrophytes such as *Canistrocarpus cervicornis* and *Zonaria tournefortii.*

**Fig 3:**
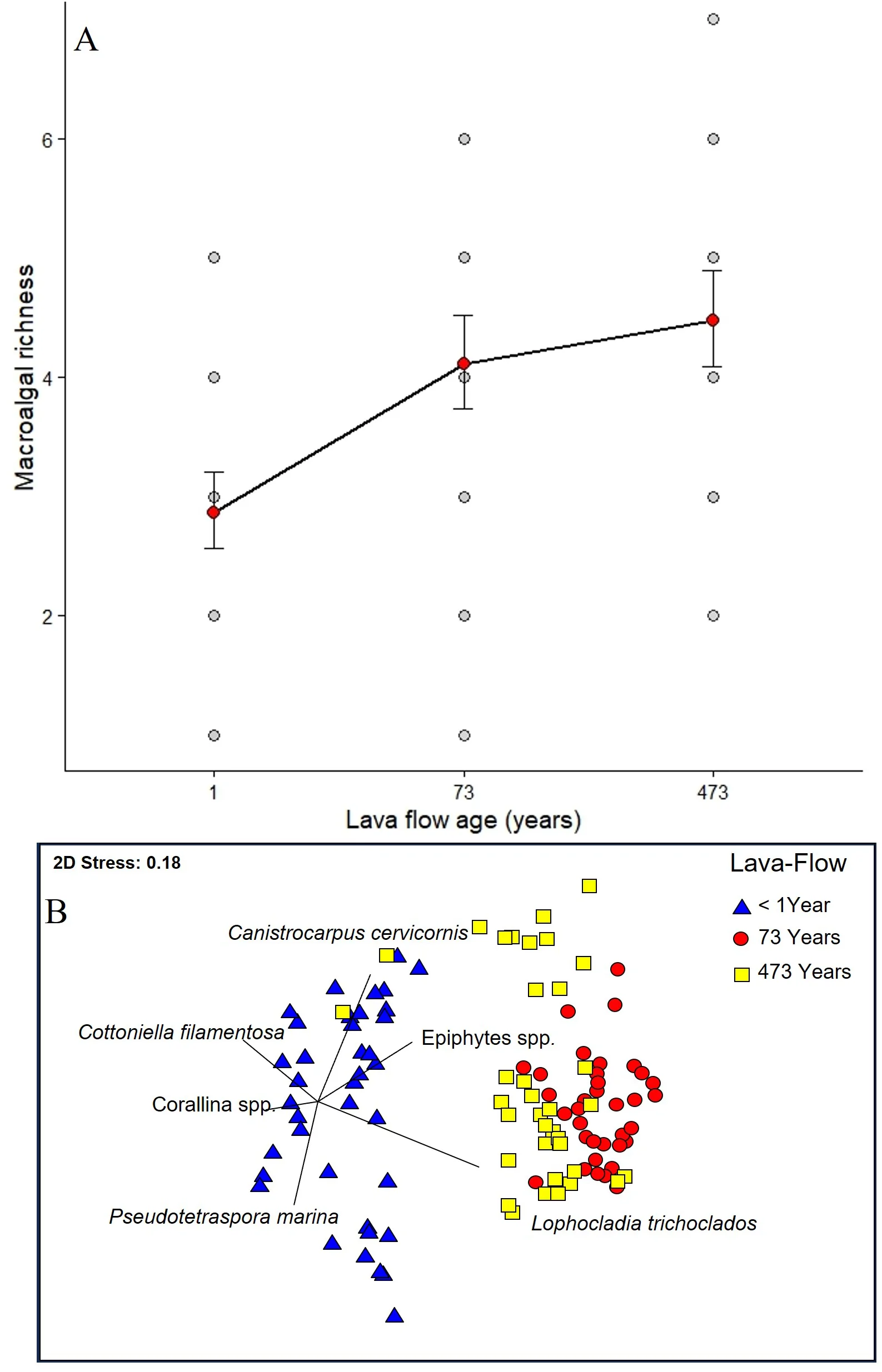
Macroalgae community structure and composition across volcanic substrates of different ages (<1 year, 73 years, 473 years). **(A)** Observed macroalgal species richness per sample (grey circles), observed mean richness per lava flow (red circles), and predicted mean richness (black line) with 95% confidence intervals derived from a Conway–Maxwell–Poisson generalized linear model. **(B)** Non-metric multidimensional scaling (nMDS) ordination based on Bray–Curtis similarity of square-root transformed percent cover data, illustrating differences in macroalgal community composition among lava flows.

Macroalgal richness differed significantly among lava flows of different ages. Generalized linear mixed models with a Conway–Maxwell–Poisson distribution revealed a strong effect of lava flow age on macroalgal richness (likelihood ratio test: χ² = 35.12, df = 2, *P* < 0.001). Post-hoc pairwise comparisons showed that richness was significantly lower on the youngest lava flow (< 1 year) compared to both the 73-year-old (*P* < 0.001) and 473-year-old flows (*P* < 0.001), whereas no significant differences were detected between the intermediate and oldest lava flows (*P* = 0.42). Predicted richness increased from the youngest lava flow to the two older lava flows, with similar predicted values for the 73- and 473-year-old substrates (Fig 3A; Supplementary S5 Table).

Macroalgal community composition and percent cover also differed significantly among lava flows. The PERMANOVA showed a significant effect of lava flow age on macroalgal assemblages (PERMANOVA, F = 7.1728, P = 0.0047; Supplementary S6 Table), suggesting that succession is accompanied by shifts in species identity and abundance indicating that community differences were primarily driven by the age of the substrate.

Pairwise comparisons revealed significant differences in macroalgal composition between the youngest flow and both the 73-year (post-hoc pairwise test, t = 3.3667, P = 0.0317) and 473-year flows (post-hoc pairwise test, t = 2.4162, P = 0.0292). In contrast, assemblages on the 73- and 473-year flows did not differ significantly (post-hoc pairwise test, t = 1.4618, P = 0.1496). These patterns are visualized in the non-metric multidimensional scaling (nMDS) plot (Fig 3B), where samples from each flow cluster separately, particularly distinguishing the youngest flow from the other two.

SIMPER analysis reflect that the greatest compositional dissimilarity occurred between the youngest and 73-year-old lava flows (SIMPER, average dissimilarity = 81.7%). This contrast was mainly driven by the higher abundance of the algae *Lophocladia trichoclados* and epiphytic taxa in the intermediate flow, which were nearly absent on the youngest substrate. Comparisons between the youngest and oldest flows also showed high dissimilarity (SIMPER, average dissimilarity = 74.43%), with increased abundance of *L. trichoclados, Canistrocarpus cervicornis, Cottoniella filamentosa*, and *Pseudotetraspora marina* in the 473-year assemblage. The comparison between the 73- and 473-year flows revealed the lowest dissimilarity (SIMPER, average dissimilarity = 50.59%), reflecting a more mature and compositionally similar community. Slight shifts in abundance explained this difference, with moderate reductions in *L. trichoclados* and epiphytes and increases in *C. cervicornis* and *P. marina* in the oldest lava flow.

### 3.2 Microinvertebrates early-stage settlement

Early-stage microinvertebrates assemblages were mainly composed of small crustaceans, polychaetes and gastropods. The youngest lava flow showed a simpler assemblage dominated by generalist early settlers, whereas the two older flows contained a broader set of taxa, including amphipods, decapods, gastropods, and polychaetes. This descriptive pattern was then evaluated statistically through richness models and multivariate analyses.

**Fig 4:**
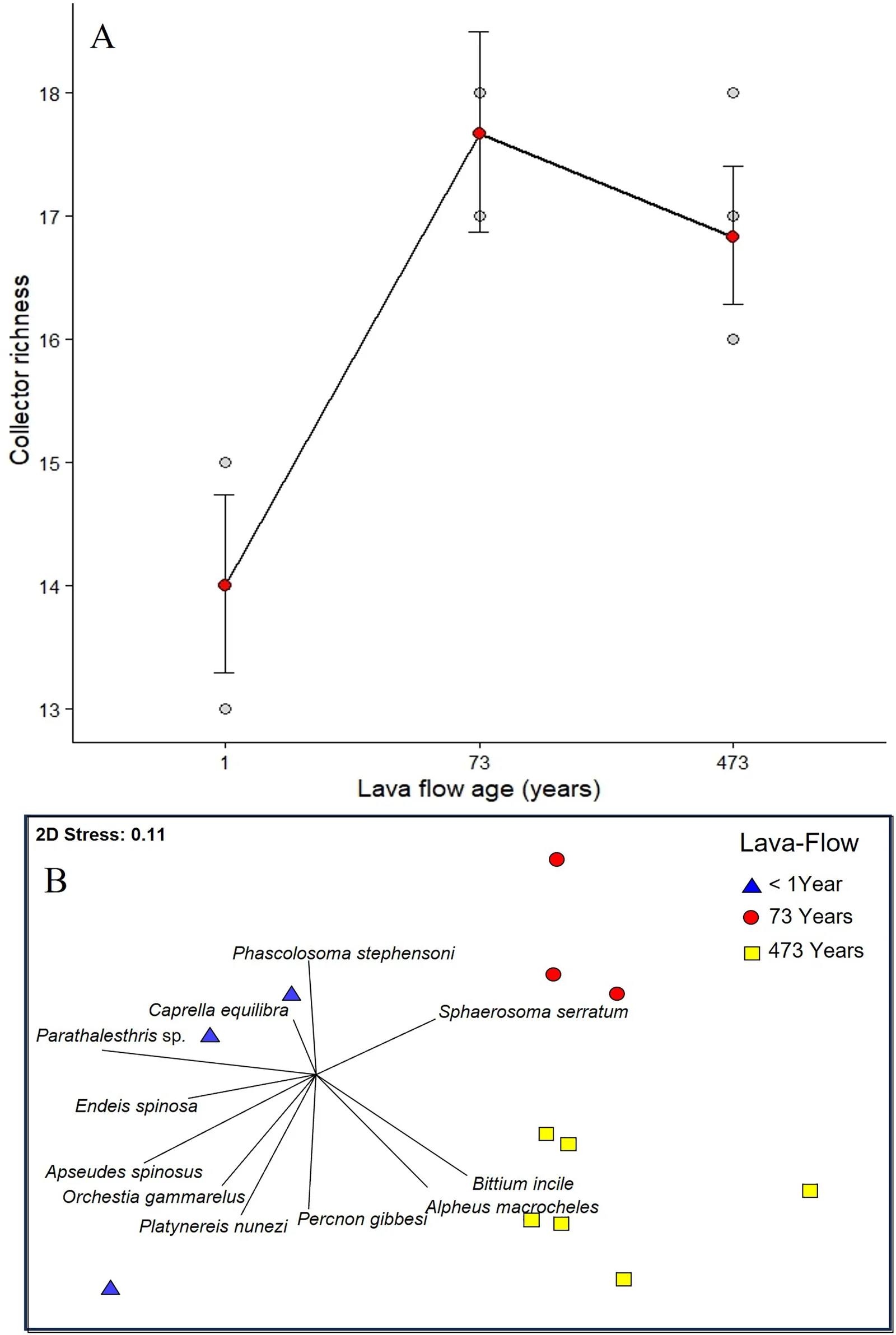
Early settled microinvertebrate across volcanic substrates of different ages (<1 year, 73 years, 473 years). **(A)** Observed species richness per sample (grey circles), observed mean richness per lava flow (red circles), and predicted mean richness (black line) with 95% confidence intervals derived from a Conway–Maxwell–Poisson generalized linear model. **(B)** Non-metric multidimensional scaling (nMDS) ordination of collector assemblages based on Bray–Curtis similarity, illustrating differences in community composition among lava flows.

Species richness of early invertebrate settlers differed significantly among lava flows of different ages (COM-Poisson GLM, likelihood ratio test: χ² = 19.77, df = 2, P < 0.0001; Fig 4A; Supplementary S7 Table). Richness was lowest on the youngest lava flow (<1 year) and increased on the older substrates. Pairwise comparisons revealed that richness in the youngest flow was significantly lower than in both the 73-year-old (P < 0.0001) and the 473-year-old flows (P < 0.0001), whereas no significant differences were detected between the intermediate and oldest flows (P = 0.22).

The analysis of early settler abundances revealed significant compositional differences across flows (PERMANOVA: F = 5.1545, P = 0.0002; Supplementary S6 Table). Pairwise tests indicated significant differences between the youngest flow and both the 73-year-old (post-hoc pairwise test, t = 1.9361, P = 0.0452) and 473-year-old (post-hoc pairwise test, t = 2.4646, P = 0.0036) flows, as well as between the two older flows (post-hoc pairwise test, t = 2.2833, P = 0.0052). These patterns are visually supported by the nMDS plot (Fig 4B), which shows partial overlap but also distinct clustering by flow age.

The highest dissimilarity was observed between the 1-year and 473-year-old flows (SIMPER, average dissimilarity = 28.93%), mainly due to reduced abundances of the copepod Thalestridae sp. and the tanaidacean *Apseudes spinosus* in the older assemblage, and increased representation of the decapod *Alpheus macrocheles*, the gastropod *Bittium incile*, and the amphipod *Caprella equilibra*. Similarly, the 1-year vs. 73-year comparison showed an average dissimilarity of 27.95%, driven by comparable taxonomic shifts, including a decrease in Thalestridae sp. and *A. spinosus*, and increases in *C. equilibra*, the isopod *Sphaerosoma serratum*, and the gastropod *B. incile*. The 73- vs. 473-year comparison yielded the lowest dissimilarity (SIMPER, average dissimilarity = 23.35%), reflecting more gradual compositional changes such as increased abundance of *A. macrocheles* and the polychaeta *Brania articulata* in the oldest flow, along with subtler variations across *C. equilibra*, *S. serratum*, the polychaeta *Phascolosoma stephensoni* and the pycnogonid *Endeis spinosa*.

### 3.3 Microinvertebrates benthic community

Established rock-associated microinvertebrate assemblages included gastropods, polychaetes, amphipods, decapods, echinoderms, bryozoans, and sipunculids. The intermediate-aged lava flow supported the richest assemblage and included several taxa absent or less frequent in the youngest and oldest flows, whereas the oldest substrate showed a more reduced but compositionally distinct assemblage. These patterns were further assessed using richness models, PERMANOVA, nMDS, and SIMPER analyses.

**Fig 5:**
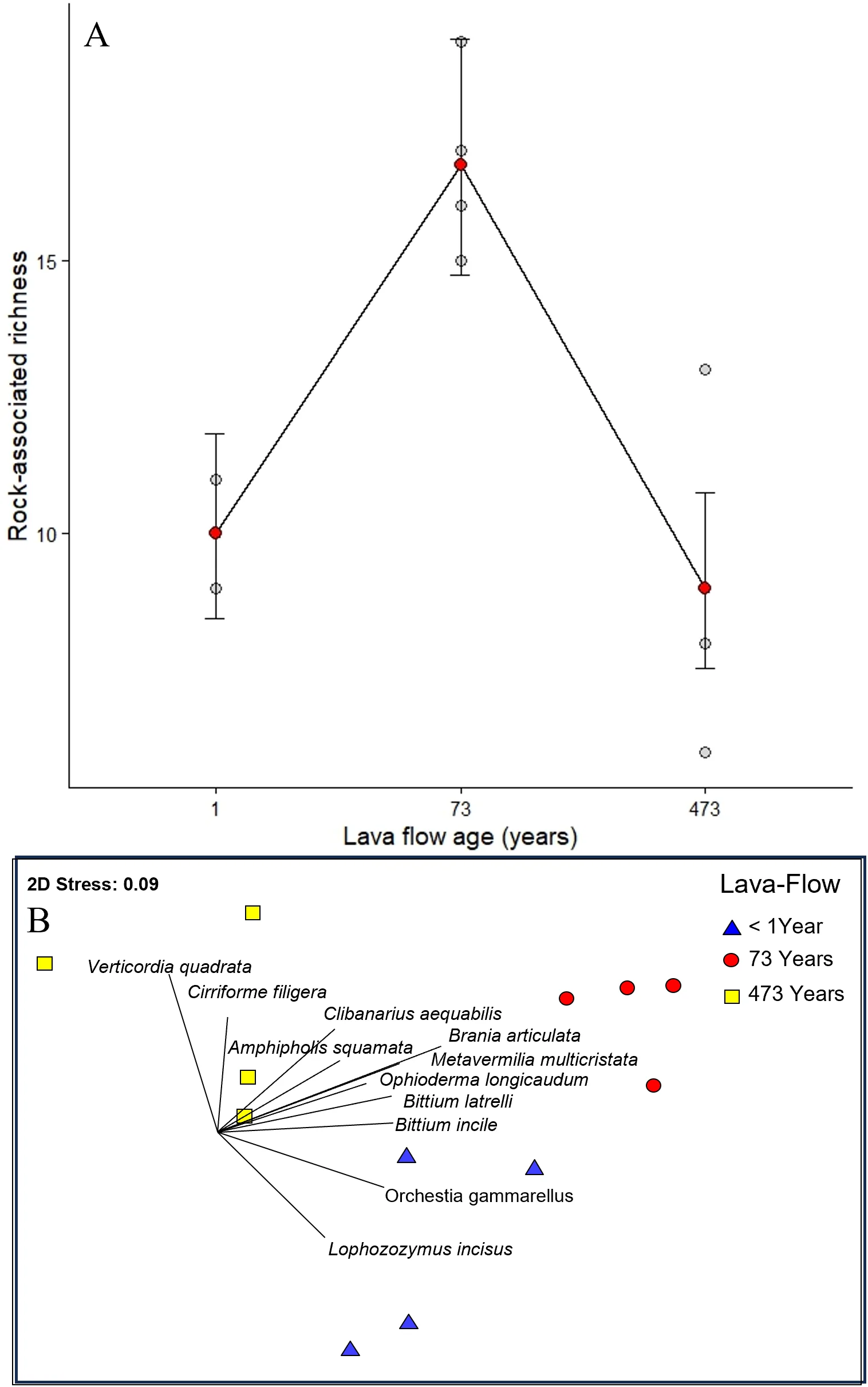
Benthic microinvertebrate communities across volcanic substrates of different ages (<1 year, 73 years, 473 years). **(A)** Observed species richness per sample (grey circles), observed mean richness per lava flow (red circles), and predicted mean richness (black line) with 95% confidence intervals derived from a Conway–Maxwell–Poisson generalized linear model. **(B)** Non-metric multidimensional scaling (nMDS) ordination based on Bray–Curtis similarity of square-root transformed abundance data, showing compositional differences among lava flows.

Species richness of sessile and slow-moving benthic microinvertebrates differed significantly among lava flows of different ages (COM-Poisson GLM, likelihood ratio test: χ² = 17.51, df = 2, P < 0.001; Fig 5A; Supplementary S8 Table). Richness peaked on the intermediate-aged lava flow (73 years), indicating a unimodal pattern along the successional gradient. Pairwise comparisons showed that richness at the 73-year-old flow was significantly higher than at both the youngest (<1 year; P < 0.0001) and the oldest (473 years; P < 0.0001) lava flows. In contrast, no significant difference was detected between the youngest and oldest flows (P = 0.67).

In terms of community composition, PERMANOVA results showed that “lava flow age” had a significant effect on the structure of microinvertebrate assemblages (PERMANOVA: F = 3.7913, P = 0.0009; Supplementary S6 Table). Pairwise comparisons suggest significant differences between all lava flow pairs: < 1 vs. 73 years (post-hoc pairwise test, t = 2.1512, P = 0.0427), < 1 vs. 473 years (post-hoc pairwise test, t = 1.9422, P = 0.0364), and 73 vs. 473 years (post-hoc pairwise test, t = 2.5693, P = 0.0371). These patterns were supported by the nMDS plot (Fig 5B), which visually highlighted distinct clustering of samples according to flow age.

The highest average dissimilarity was found between the 73- and 473-year-old flows (SIMPER, average dissimilarity = 49.38%).This contrast was mainly driven by a sharp reduction in the abundance of two species of gastropods, namely *Bittium incile, Bittium latrelli,* and polychaetes such as *Metavermilia multicristata*, and *Brania articulata*, and along with the loss of several taxa present only in the 73-year assemblage, such as two representative of the order Ophiurida (*Ophioderma longicaudum*, *Amphipholis squamata*) and the amphipod *Caprella equilibra*. The comparison between the youngest and the 473-year-old flows (SIMPER, average dissimilarity = 44.38%) revealed a similar pattern: early colonizers like *M. multicristata*, *B. latrelli* and the amphipod *Orchestia gammarelus* declined, while the Bivalvia *Verticordia quadrata* and the endolithic sessile polychaetes as *Cirriformia filigera* became more prominent. Lastly, dissimilarity between the youngest and the 73-year-old flows (SIMPER, average dissimilarity = 41.76%) still reflected clear successional turnover, marked by greater abundance and richness in the intermediate flow, including several species absent from the youngest site, such as *O. longicaudum*, *A. squamata*, and the hermit-crab *Clibanarius aequabilis*.

### 3.4. Successional pattern

**Fig 6:**
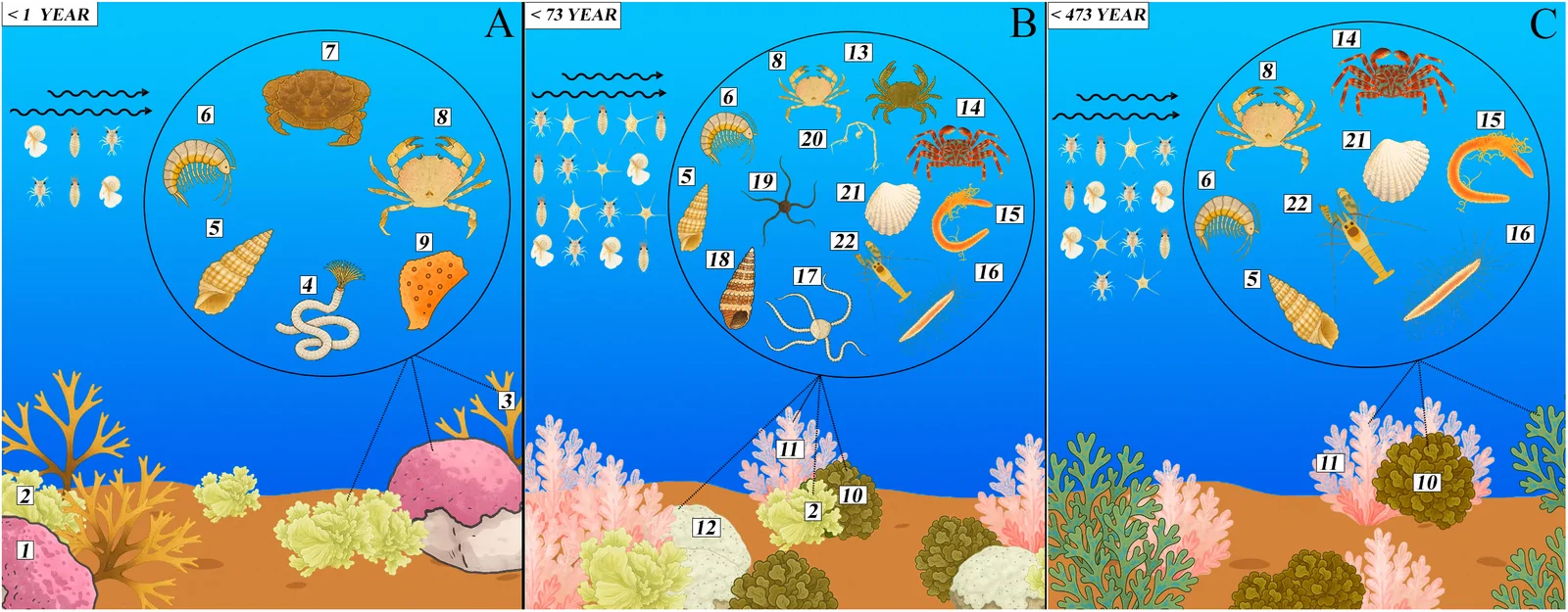
Infographic comparing benthic community composition across three volcanic lava flows of different ages (A, < 1 year; B, 73 years; C, 473 years) on La Palma Island. Each panel illustrates representative macroalgal species and successfully settled invertebrates observed on the corresponding substrate. Species: ***1**- Corallina* spp., ***2**- Pseudotetraspora marina*, ***3**- Canistrocarpus cervicornis*, ***4**- Metavermilia multicristata*, ***5**- Bittium latreillii*, ***6**- Orchestia gammarellus*, ***7**- Xantho poressa*, ***8**- Lophozozymus incisus*, ***9**- Crisisa* sp., ***10**- Zonaria tourneforti*, ***11**- Lophocladia trichoclados*, ***12**- Porolithon onkodes*, ***13**- Pachygrapsus marmoratus*, ***14**- Percnon gibbesi*, ***15**- Cirriformia filigera*, ***16**- Brania articulata*, ***17**- Amphipholis squamata*, ***18**- Bittium incile*, ***19**- Ophioderma longicauda*, ***20**- Caprella equilibra*, ***21**- Verticordia quadrata*, ***22**- Alpheus macrocheles*, ***23**- Dictyota humifusa*.

The comparison of the three lava flows revealed distinct shifts in species composition across the selected benthic components analysed: macroalgal assemblages, early post-settlement microinvertebrates, and established rock-associated microinvertebrates. These patterns suggest a progressive successional gradient, although the strength and shape of the response differed among biological components. The taxa highlighted in this section and in Fig 6 were selected as representative examples because they were among the dominant taxa within each lava flow and/or contributed substantially to compositional differences among lava flows according to the SIMPER analyses. The complete taxonomic dataset is provided in S1 and S2 Tables; here, we summarize the most abundant, dominant, or discriminant taxa to facilitate interpretation of the successional pattern. In the youngest lava flow (<1 year), benthic communities included among the macroalgae *Cottoniella filamentosa*, *Canistrocarpus cervicornis*, *Pseudotetraspora marina*, and *Corallina* spp. The early invertebrate settlers comprised Polychaeta as *Brania articulata* and *Sabella spallanzanii*, amphipods as *Orchestia gammarellus and Amphitoe rubricata*, also tanaidaceans as *Apseudes spinosus*, and brachyurans (*Lophozozymus incisus, Xantho poressa*). The benthic microinvertebrate community was characterized by Gastropods as *Bittium latrelli*, Polychaeta like *Metavermilia multicristata*, and some Amphipods as *O. gammarellus and A. rubricata* (Fig 6A).

The intermediate lava flow (73 years) supported the highest species richness and taxonomic distinctiveness mainly for established rock-associated microinvertebrates, while macroalgal assemblages and early post-settlement assemblages showed greater similarity between the intermediate and oldest substrates. The macroalgal assemblage included *Lophocladia trichoclados*, *Porolithon onkodes*, *Zonaria tournefortii*, and various epiphytic species. Early invertebrate settlers in this flow showed higher diversity including amphipods as *Caprella equilibra* and *O. gammarellus*, the decapod *Alpheus macrocheles*, the Gastropod *Bittium incile*, and the Polichaete *Brania articulata*. Benthic microinvertebrates included *B. incile*, *B. latrelli*, *B. articulata*, and unique taxa such as the sessile endolytic polychaete *Cirriformia filigera*, the brachyura *Pachygrapsus marmoratus*, and three species of echinoderms, *Paracentrotus lividus*, *Amphipholis squamata*, and *Ophioderma longicaudum* (Fig 6B).

In the oldest lava flow (473 years), the macroalgal community included *Dictyota humifusa*, *Zonaria tournefortii*, *Lophocladia trichoclados*, and *Porolithon onkodes*. Early settlers included *A. macrocheles*, *B. incile*, and *B. articulata*, with reduced abundance of Thalestridae sp*.* and *A.spinosus*. The benthic microinvertebrate community was dominated by *Verticordia quadrata*, *B. articulata*, *B. incile*, *B. latrelli*, *C. filigera*, *M. multicristata*, and *A. macrocheles*. Additional taxa recorded as larvae, juveniles, early settlers, or small cryptic individuals, such as *Percnon gibbesi*, *Macropodia longirostris* and *Clibanarius aequabilis*, were also present (Fig 6C).

Overall, the successional trajectory observed across the lava flows was not identical for all biological components. Macroalgal assemblages showed a marked contrast between the youngest substrate and the two older flows, with no significant differences between the intermediate and oldest lava flows, whereas established rock-associated microinvertebrates showed a clearer intermediate-stage richness peak and stronger compositional turnover across the full gradient.

## 4. Discussion

The temporal gradient of lava flow age provided a natural framework to explore how selected components of shallow benthic communities assemble over time following volcanic disturbances. Our findings show that the response to lava-flow age differed among biological components. Macroalgal assemblages showed a strong contrast between the youngest flow and the two older substrates, suggesting relatively early compositional stabilization after the initial colonization stage. In contrast, established rock-associated microinvertebrates exhibited a clearer peak in richness at the intermediate-aged flow and significant compositional turnover among all lava flows. Early post-settlement microinvertebrates showed an intermediate pattern, with richness increasing from the youngest substrate to the two older flows, while community composition continued to differ among lava flows (Fig 6). Thus, rather than indicating a uniform successional response across the benthic community components under study, our results suggest component-specific trajectories within shallow benthic assemblages developing on volcanic rocky substrates. These patterns are consistent with a successional trajectory in which initial colonization is followed by habitat-mediated diversification and, particularly among established microinvertebrates, increasing community structuring over time [15,16].

In this study, lava-flow age was the main factor considered to describe differences among the benthic components analysed. The close spatial proximity of the three lava flows, their similar coastal orientation, and their comparable sampling depth likely reduced major environmental differences among sites. In addition, all samples were collected during a single summer survey in August 2022, and the assemblages recorded here should therefore be interpreted as a temporally standardized snapshot rather than as year-round patterns. Many macroalgae and small benthic invertebrates may show seasonal dynamics, short life cycles, or recruitment pulses, and interannual variability or anomalous oceanographic conditions may influence colonization, settlement, and community composition. However, all lava flows were sampled during the same period, at comparable depth, and along the same coastal sector, allowing meaningful comparisons among substrates of contrasting ages under the same seasonal conditions. Future seasonal and interannual surveys would be needed to determine the temporal consistency of the successional patterns described here.

Comparable studies in other volcanic marine systems remain scarce, particularly for small benthic invertebrates and early post-settlement assemblages. Nevertheless, available evidence from volcanic disturbances such as the Tagoro submarine eruption in El Hierro, the Kasatochi eruption in Alaska, and early succession on Vailulu’u seamount indicates that newly formed volcanic substrates are often colonized rapidly by opportunistic or highly dispersive taxa, followed by progressive changes in community composition as habitat structure and biotic interactions develop [3,6]. Our results are consistent with this broader pattern, but also show that different benthic components may follow distinct successional trajectories, with macroalgal assemblages stabilizing earlier than established rock-associated microinvertebrates. This highlights the value of incorporating small benthic organisms and early settlement stages into studies of post-eruption marine succession.

### 4.1 Successional patterns in macroalgal assemblages

Macroalgae are among the earliest colonizers of newly formed volcanic lava flows, initiating benthic community development, as observed by Sangil et al. (2024). Their contribution to substrate stabilization and trophic support makes them essential for the establishment of subsequent invertebrate communities [39, 40]. Although the dominant species identified differ between our study and Sangil et al. [13], both coincide in showing that early colonization was led by fast-growing opportunistic algae. These discrepancies may reflect the fact that the surveys were conducted during different phases of the very early succession, as well as the strong microhabitat heterogeneity of the lava substrate. In addition, methodological differences are relevant, since our study focused on 10–20 m depth whereas Sangil et al. [13] covered the full range from the intertidal to 20 m. These factors, along with possible seasonal variability in opportunistic or ephemeral macroalgae, may help explain the taxonomic differences observed while reinforcing the functional consistency of early successional patterns. Among the species detected in our survey, several pioneer macroalgae exhibited traits associated with early successional success, including physiological tolerance, rapid growth, and effective substrate attachment. These traits are well documented in opportunistic red, brown, and green macroalgae inhabiting recently disturbed volcanic substrates [41,42]. For example, fast-growing filamentous red macroalgae such as *Asparagopsis taxiformis* and *Cotoniella filamentosa* are known for their high physiological plasticity and genetic adaptability, traits that confer resilience under the variable and stressful conditions typical of post-eruption environments [43,44]. *Canistrocarpus cervicornis*, characterized by high dispersal and colonization capacity, was among the earliest settlers and remained abundant across all successional stages, consistent with its widespread occurrence in the Canary Islands [45,46].

These early functional advantages set the stage for subsequent changes in community composition. As substrates mature and become progressively more stable and biologically enriched, benthic communities tend to converge towards more complex and diverse macroalgal assemblages. Accordingly, the 73- and 473-year-old flows exhibited high similarity in macroalgal composition, suggesting that many of the structural and ecological conditions required for macroalgal development were already established by the intermediate successional stage. In contrast, the absence of several taxa in the youngest flow likely reflects the limited substrate stability and lower biological conditioning typical of early post-eruption environments [2,16]

In early successional stages, pioneer species dominate due to their capacity to colonize unstable substrates under minimal competition [2,15,16,24]. As substrate stability increases and organic matter accumulates, conditions become more suitable for macroalgae with higher metabolic and structural requirements, such as *Dictyota humifusa* and *Sargassum* sp. [25]. Chemical analyses following the 2021 Tajogaite eruption revealed elevated iron concentrations in coastal waters, enhancing nutrient availability and potentially facilitating macroalgal colonization during subsequent successional stages [12].

Progressive substrate stabilization also likely enabled the establishment of calcifying coralline algae with specific substrate requirements, such as *Porolithon onkodes*. This species is typically associated with biologically conditioned substrates and contributes to long-term benthic consolidation through reef-building and calcification processes [47]. Although limitations in taxonomic resolution for early coralline pioneers in the youngest flow preclude a detailed assessment of competitive or facilitative interactions, the delayed appearance of *P. onkodes* is consistent with its reliance on stabilized substrates.

Similarly, the occurrence of *Zonaria tournefortii* and epiphytic algae reflects a more structurally complex community in which interspecific interactions become increasingly important at later successional stages [48]. Overall, these patterns align with classical models of marine benthic succession, where increasing substrate stability and habitat complexity promote higher species richness and more specialized assemblages over time [16,49].

### 4.2 Colonization dynamics of early microinvertebrates on settlement collectors

The distinct macroalgal assemblages characterizing each successional stage may have contributed to shaping the composition of early invertebrate colonizers. Beyond their role as primary settlers, macroalgae can mediate invertebrate recruitment through habitat structure and surface properties that facilitate or inhibit settlement [50,51]. Across all lava flows, generalist omnivorous and detritivorous crustaceans, such as the hermit crab *Clibanarius aequabilis* and amphipods (e.g., *Orchestia gammarellus*), were consistently present, reflecting broad trophic plasticity and tolerance to early successional conditions.

In the youngest lava flow, structurally simple and fast-growing algae favored early settlement by mobile generalist crustaceans, including brachyuran crabs (e.g., *Xantho poressa*) and small detritivores (e.g., *Apseudes spinosus*) [52]. The intermediate-aged flow supported a broader functional spectrum of invertebrates, including grazers and predators such as the echinoid *Arbacia lixula* and the decapod *Cronius ruber*, likely promoted by increased algal structural complexity. In this stage, calcifying algae such as *Porolithon onkodes*, together with branched macroalgae (*Lophocladia trichoclados*, *Zonaria tournefortii*), enhanced habitat complexity and substrate stability, supporting more diverse assemblages [53,54].

In the oldest lava flow, community structure was dominated by competitively strong and behaviorally specialized decapods, including the snapping shrimp *Alpheus macrocheles* and the rock crab *Percnon gibbesi*, taxa known to thrive under intense competition and predation pressure [16,55]. Although calcifying algae were less predominant at this stage, they likely continued to contribute to substrate consolidation, maintaining suitable attachment surfaces for late-successional invertebrates [56].

These habitat-mediated dynamics appear to follow different temporal trajectories for macroalgal and invertebrate communities. Our results suggest that macroalgal assemblages may reach a compositional equilibrium earlier, as no significant differences were detected between the 73- and 473-year-old flows. In contrast, invertebrate communities exhibited distinct shifts across all stages, suggesting a slower and more variable stabilization process likely influenced by habitat complexity and macroalgal structure. Species richness peaked in the intermediate-aged lava flow, which also supported a broader range of functional traits. This included pioneer taxa typically associated with early colonization and r-selected strategies, as well as more specialized organisms adapted to mature environments. These patterns underscore the transitional importance of intermediate successional stages, where both colonization opportunities and competitive interactions shape community assembly. However, the different trajectories observed for macroalgae and invertebrates indicate that these components are not necessarily tightly coupled through time. Although macroalgal structure can facilitate mobile epifauna by increasing habitat complexity, food availability, and refuge availability, associated invertebrates may also respond to additional factors such as larval supply, dispersal capacity, predation, competition, and the availability of cryptic microhabitats. This is consistent with previous studies showing that macroalgal complexity can influence epifaunal assemblages, but that faunal responses may vary depending on the identity, architecture, and temporal stability of the algal habitat [40,50,52].

Understanding the functional contributions of different taxa within this framework is essential to elucidate how benthic communities expand and stabilize over time. The role of macroalgae as primary settlers extends beyond their contribution to substrate stabilization and nutrient cycling. By increasing habitat complexity, they also create essential microhabitats that facilitate the colonization of benthic invertebrates [40]. Small benthic invertebrate communities are typically associated with rocky substrates and various algal species, comprising a diverse array of small invertebrates from multiple taxonomic groups. It includes arthropods, such as isopods, amphipods, and tanaidaceans, as well as small decapods, mollusks, polychaetes, and some echinoderms [57]. Although these taxonomic groups are common in many benthic habitats, their presence in the youngest and intermediate lava flows is ecologically relevant because small, mobile, and opportunistic invertebrates are often among the first organisms able to exploit newly available volcanic substrates and associated microhabitats, as also reported after the Kasatochi eruption, Alaska, USA [3].

### 4.3 Structure of established benthic microinvertebrate communities

Among early colonizers, small grazing gastropods exemplify the functional traits that facilitate rapid establishment on newly formed substrates. Species such as *Bittium incile* illustrate high trophic flexibility and efficient exploitation of biofilms and early algal growth, traits commonly associated with successful settlement in post-disturbance habitats [58]. Early assemblages also include suspension-feeding bivalves, whose generalist feeding modes and protective shells can promote persistence under unstable conditions, as observed for *Verticordia quadrata* [58]. In addition, some brachyuran crabs represent mobile pioneer predators; taxa such as *Lophozozymus incisus* exemplify physiological tolerance and behavioral plasticity that may favor colonization of dynamic nearshore environments following volcanic disturbance [22,23]. For other taxa such as the syllid annelid *Brania articulata*, and amphipods *Orchestia gammarellus* and *Amphitoe rubricata*, specific competitive advantages are less documented; however, their functional traits offer insight. These groups often rely on detritus or suspended particles as primary food sources [59], and their trophic plasticity may facilitate rapid colonization of nutrient-rich but structurally simple habitats. Amphipods can function as detritivores or filter feeders, whereas syllids may exploit detrital resources; these trophic traits can support their early establishment on freshly formed substrates [60–62].

As the substrate matures, greater species richness and the emergence of exclusive taxa appear to result from improved habitat complexity and resource availability [63]. However, in later successional stages, competitive interactions likely intensify, potentially leading to declines in diversity through niche filtering or exclusion, as predicted by classical successional theory [16]. In the most mature community, we observed a shift towards species exhibiting traits of competitive dominance and niche specialization, such as the decapod *Alpheus macrocheles* that is a predator known for its asymmetrical snapping claw, which produces a powerful cavitation bubble used to stun prey a trait that enhances both foraging efficiency and defense [64]. Similarly, *Macropodia longirostris* exemplifies cryptic adaptation; its capacity to camouflage within algae and substrates not only reduces predation risk but also improves its success as an ambush predator [65]. In addition, the consistent presence and dominance of the crab *Percnon gibbesi* in the oldest flow may reflect its well-documented competitive strength. Originally introduced into the Mediterranean, where it is considered an invasive species, *P. gibbesi* has demonstrated high adaptability, environmental tolerance, and success in outcompeting native species in mature rocky environments [66,67]. In addition to these mobile taxa, the presence of sediment-dependent or endolithic polychaetes as *Cirriformia filigera* further reflects the structural maturation of the substrate. These organisms require consolidated surfaces and the accumulation of fine sediments to construct protective tubes or burrows, indicating increased habitat stability and microhabitat availability in the oldest lava flow. Its ability to establish and thrive in the complex, resource-saturated habitat of the 473-year-old lava flow suggests that similar competitive advantages may be at play in this system.

These observations raise broader ecological questions regarding the trajectory and stability of successional outcomes in volcanic marine environments. Although our sampling design does not directly test post-settlement filtering experimentally, additional indirect insight emerges when comparing taxa recorded in settlement collectors with those recorded in established rock-associated assemblages across the three lava flows. In the youngest flow, the number of settled taxa was slightly lower than the number of larval arrivals, suggesting a modest degree of post-settlement filtering. In the intermediate-aged flow (73 years), this relationship was nearly balanced, indicating that habitat conditions were generally favorable for successful establishment. In contrast, the oldest flow (473 years) showed a marked reduction in the proportion of taxa transitioning from arrival to recruitment. This pattern is consistent with an intensification of post-settlement selection pressures as communities mature, potentially associated with increased competition, predation, and habitat structuring.

Although not a primary objective of this study, our results indicate that post-settlement filtering may intensify as benthic communities mature, highlighting the need for future research explicitly addressing seasonal recruitment success and habitat filtering in late successional stages. Comparable patterns have been reported in other marine systems, such as kelp forests, where communities may continue to diverge long after habitat stabilization due to stochastic colonization, shifting species interactions, and disturbance legacies. Together, these parallels emphasize the broader ecological relevance of investigating long-term successional dynamics in active volcanic coastal environments.

## 5. Conclusion

Volcanically generated substrates offer exceptional natural experiments to examine how marine benthic communities assemble after large-scale disturbances. By analysing lava flows spanning nearly five centuries of ecological development, our study shows that selected components of shallow benthic communities exhibit distinct successional responses. Macroalgal assemblages appeared to stabilize relatively early, with marked differences between the youngest substrate and the two older lava flows, whereas established rock-associated microinvertebrates showed a clearer richness peak at the intermediate-aged substrate and stronger compositional turnover across the chronosequence. These patterns suggest that early colonization is followed by habitat-mediated diversification, while long-term community maturation may be increasingly influenced by habitat structure, biotic interactions, and post-settlement filtering. Together, these findings underscore the importance of substrate age and habitat complexity in structuring coastal benthic assemblages and demonstrate the ecological value of volcanic coastlines as natural observatories of marine community assembly.

## Supporting information

S1 Table Taxonomic list of macroalgal species identified across the three lava flows. (DOCX)

S2 Table Taxonomic list of microinvertebrate taxa identified across the three lava flows. (DOCX)

S3 Table Residual diagnostics for generalized linear models of species richness. (DOCX)

S4 Table Preliminary analyses comparing sampling sites within lava flows. (DOCX)

S5 Table Pairwise comparisons for macroalgal species richness among lava flows. (DOCX)

S6 Table PERMANOVA and pairwise comparisons of community composition among lava flows. (DOCX)

S7 Table Pairwise comparisons for early-settler species richness among lava flows. (DOCX)

S8 Table Pairwise comparisons for benthic microinvertebrate species richness among lava flows. (DOCX)
