# Supplementary material for: Benthic communities on recent and historical volcanic lava flows of an oceanic island
Source: PLoS One. 2026 Sep 23;21(9):e0357447. doi: 10.1371/journal.pone.0357447 (PMC13600511; doi:10.1371/journal.pone.0357447)
Supplement: Supplementary file 1 [file pone.0357447.s001.docx]

**Supplementary Table S1.** Taxonomic list of macroalgal species identified in the different lava-flows.

| **Phylo** | **Class** | **Order** | **Family** | **Species** |
| --- | --- | --- | --- | --- |
| Chlorophyta | Chlorophyceae | Chlamydomonadales | Palmellopsidaceae | *Pseudotetraspora marina* Wille |
| Heterokontophyta | Phaeophyceae | Dictyotales | Dictyotaceae | *Canistrocarpus cervicornis* (Kützing) De Paula and De Clerck  *Dictyota cyanoloma* Tronholm, De Clerck, A.Gómez-Garreta & Rull Lluch |
|  |  |  |  | *Dictyota humifusa* Hörnig, Schnetter & Coppejans |
|  |  |  |  | *Zonaria tournefortii* (J.V.Lamouroux) Montagne |
|  |  | Fucales | Sargassaceae | *Sargassum* sp. (Turner) C.Agardh, |
| Rhodophyta | Florideophyceae | Bonnemaisoniales | Bonnemaisoniaceae | *Asparagopsis taxiformis* (Delile) Trevisan |
|  |  | Ceramiales | Delesseriaceae | *Cottoniella filamentosa* (M.A.Howe) Børgesen |
|  |  |  | Rhodomelaceae | *Lophocladia trichoclados*  F.Schmitz |
|  |  | Corallinales | Porolithaceae | *Porolithon onkodes* (Heydrich) Foslie |
|  |  |  | Corallinaceae | *Corallina* spp. |
|  |  | Nemaliales | Liagoraceae | *Liagora* sp. J.V.Lamouroux |
