# Supplementary material for: Benthic communities on recent and historical volcanic lava flows of an oceanic island
Source: PLoS One. 2026 Sep 23;21(9):e0357447. doi: 10.1371/journal.pone.0357447 (PMC13600511; doi:10.1371/journal.pone.0357447)
Supplement: Supplementary file 2 [file pone.0357447.s002.docx]

**Supplementary Table S2.** Taxonomic list of epifaunal invertebrate taxa identified across the lava-flows.

| Phylo | Class | Order | Family | Species |
| --- | --- | --- | --- | --- |
| Bryozoa | Stenolaemata | Cyclostomatida | Crisiidae | *Crisisa* sp. |
|  |  |  | Lichenoporidae | *Disporella* sp. |
| Mollusca | Bivalvia | Anomalodesmata | Verticordiidae | *Verticordia quadrata* E. A. Smith, 1885 |
|  | Gastropoda | Caenogastropoda | Cerithiidae | *Bittium incile* R. B. Watson, 1897 |
|  |  |  |  | *Bittium latreillii* (Payraudeau, 1826) |
| Annelida |  | Sipuncula | Phascolosomatidae | *Phascolosoma (Phascolosoma) stephensoni* (Stephen, 1942) |
|  | Polychaeta | Eunicida | Eunicidae | *Nicidion saxicola* (Langerhans, 1881) |
|  |  | Phyllodocida | Nereididae | *Platynereis nunezi* Teixeira, Ravara, …, 2022 |
|  |  |  | Syllidae | *Brania articulata* Hartmann-Schröder, 1982 |
|  |  | Sabellida | Serpulidae | *Metavermilia multicristata (Philippi, 1844)* |
|  |  | Terebellida | Cirratulidae | *Cirriformia filigera* (Delle Chiaje, 1828) |
| Arthropoda | Copepoda | Harpacticoida | Thalestridae | *Parathalestris* sp*.* |
|  | Malacostraca | Amphipoda | Ampeliscidae | *Ampelisca brevicornis* (A. Costa, 1853) |
|  |  |  | Ampithoidae | *Ampithoe rubricata* (Montagu, 1808) |
|  |  |  | Caprellidae | *Caprella equilibra* Say, 1818 |
|  |  |  | Talitridae | *Orchestia gammarellus* (Pallas, 1766) |
|  |  | Decapoda | Alpheidae | *Alpheus macrocheles* (Hailstone, 1835) |
|  |  |  | Diogenidae | *Clibanarius aequabilis* (Dana, 1851) |
|  |  |  | Grapsidae | *Pachygrapsus marmoratus* (Fabricius, 1787) |
|  |  |  | Hippolytidae | *Hippolyte inermis* Leach, 1816 |
|  |  |  | Inachidae | *Macropodia longicornis* (A. Milne-Edwards & Bouvier, 1899) |
|  |  |  | Percnidae | *Percnon gibbesi* (H. Milne Edwards, 1853) |
|  |  |  | Xanthidae | *Lophozozymus incisus* (H. Milne Edwards, 1834) |
|  |  |  |  | *Xantho poressa* (Olivi, 1792) |
|  |  | Isopoda | Sphaeromatidae | *Sphaeroma serratum* (J. C. Fabricius, 1787) |
|  |  | Tanaidacea | Apseudidae | *Apseudes spinosus* (M. Sars, 1858) |
|  | Pycnogonida | Pantopoda | Endeidae | *Endeis spinosa* (Montagu, 1808) |
| Echinodermata | Echinoidea | Arbacioida | Arbaciidae | *Arbacia lixula* (Linnaeus, 1758) |
|  |  | Camarodonta | Parechinidae | *Paracentrotus lividus* (Lamarck, 1816) |
|  |  |  | Toxopneustidae | *Sphaerechinus granularis* (Lamarck, 1816) |
|  | Ophiuroidea | Amphilepidida | Amphiuridae | *Amphipholis squamata* (Delle Chiaje, 1828) |
|  |  | Ophiacanthida | Ophiodermatidae | *Ophioderma longicaudum* (Bruzelius, 1805) |
