# Supplementary material for: Benthic communities on recent and historical volcanic lava flows of an oceanic island
Source: PLoS One. 2026 Sep 23;21(9):e0357447. doi: 10.1371/journal.pone.0357447 (PMC13600511; doi:10.1371/journal.pone.0357447)
Supplement: Supplementary file 3 [file pone.0357447.s003.docx]

**Supplementary Table S3.** Simulation-based residual diagnostics for Conway–Maxwell–Poisson generalized linear models fitted to species richness across lava flows for macroalgae, early-stage settlers (collectors), and benthic microinvertebrates (rocks). Model assumptions were evaluated using DHARMa (1000 simulations), including tests for residual uniformity (Kolmogorov–Smirnov test), dispersion, and outliers. Non-significant p-values indicate no evidence of model misspecification, overdispersion/underdispersion, or outlier inflation, supporting the adequacy of the fitted models.

| Biological group | n | (KS) D | Uniformity p-value | Dispersion p-value | Outliers detected | Outliers p-value | 95% CI |
| --- | --- | --- | --- | --- | --- | --- | --- |
| Macroalgae | 110 | 0.078 | 0.506 | 0.943 | 0 | 1.000 | 0-0 |
| Collectors | 12 | 0.192 | 0.697 | 1.061 | 0 | 1.000 | 0-0 |
| Rocks | 12 | 0.210 | 0.590 | 0.961 | 0 | 1.000 | 0-0 |
