# Supplementary material for: Benthic communities on recent and historical volcanic lava flows of an oceanic island
Source: PLoS One. 2026 Sep 23;21(9):e0357447. doi: 10.1371/journal.pone.0357447 (PMC13600511; doi:10.1371/journal.pone.0357447)
Supplement: Supplementary file 4 [file pone.0357447.s004.docx]

**Supplementary Table S4.** Results of preliminary tests evaluating differences between sampling sites within lava flows.

| **Group** | **Response** | **Test** | **Statistic** | **P-value** |
| --- | --- | --- | --- | --- |
| Macroalgae | Richness | COM-Poisson GLM | χ² = 0.84 | P = 0.36 |
| Macroalgae | Composition | PERMANOVA | Pseudo-F = 0.91 | P = 0.58 |
| Early settlers | Richness | COM-Poisson GLM | χ² = 0.47 | P = 0.49 |
| Early settlers | Composition | PERMANOVA | Pseudo-F = 1.02 | P = 0.41 |
| Benthic microinv. | Richness | COM-Poisson GLM | χ² = 0.62 | P = 0.43 |
| Benthic microinv. | Composition | PERMANOVA | Pseudo-F = 0.88 | P = 0.61 |
