# Supplementary material for: Benthic communities on recent and historical volcanic lava flows of an oceanic island
Source: PLoS One. 2026 Sep 23;21(9):e0357447. doi: 10.1371/journal.pone.0357447 (PMC13600511; doi:10.1371/journal.pone.0357447)
Supplement: Supplementary file 5 [file pone.0357447.s005.docx]

**Supplementary Table S5.** Results of the Conway–Maxwell–Poisson generalized linear model (COM-Poisson GLM) assessing differences in macroalgal species richness among lava flows of different ages (<1 year, 73 years, 473 years).

| (A) **Model coefficients (COM-Poisson GLM)** | | | | |
| --- | --- | --- | --- | --- |
| Term | Estimate | SE | Z value | P-value |
| 1 Year | 1.0538 | 0.0574 | 18.35 | < 0.001 |
| 73 Years | 0.3599 | 0.0751 | 4.79 | < 0.001 |
| 473 Years | 0.4441 | 0.0737 | 6.02 | < 0.001 |
| **(B) Global effect of lava flow age** | | | | |
| Test | | χ² | df | P-value |
| Likelihood ratio test | | 35.12 | 2 | < 0.001 |
| **(C) Tukey-adjusted pairwise comparisons** | | | | |
| Comparison | | Estimate | z value | P value |
| 1 vs 73 years | | −0.3599 | −4.79 | < 0.001 |
| 1 vs 473 years | | −0.4441 | −6.02 | < 0.001 |
| 73 vs 473 years | | −0.0842 | −1.26 | 0.42 |
