# Supplementary material for: Benthic communities on recent and historical volcanic lava flows of an oceanic island
Source: PLoS One. 2026 Sep 23;21(9):e0357447. doi: 10.1371/journal.pone.0357447 (PMC13600511; doi:10.1371/journal.pone.0357447)
Supplement: Supplementary file 6 [file pone.0357447.s006.docx]

**Supplementary Table S6.** PERMANOVA results testing the effect of lava flow age on community composition (abundance data).

| **Group** | **Factor** | **df** | **Pseudo-F** | **P (perm)** |
| --- | --- | --- | --- | --- |
| Macroalgae | Lava flow age | 2 | 7.17 | 0.0047 |
| Early settlers | Lava flow age | 2 | 5.15 | 0.0002 |
| Benthic microinvertebrates | Lava flow age | 2 | 3.79 | 0.0009 |
