# Supplementary material for: Benthic communities on recent and historical volcanic lava flows of an oceanic island
Source: PLoS One. 2026 Sep 23;21(9):e0357447. doi: 10.1371/journal.pone.0357447 (PMC13600511; doi:10.1371/journal.pone.0357447)
Supplement: Supplementary file 7 [file pone.0357447.s007.docx]

**Supplementary Table S7.** Results of the Conway–Maxwell–Poisson generalized linear model (COM-Poisson GLM) evaluating differences in species richness of early invertebrate settlers among lava flows of different ages (<1 year, 73 years, 473 years).

| (A) **Model coefficients (COM-Poisson GLM)** | | | | |
| --- | --- | --- | --- | --- |
| Term | Estimate | SE | Z value | P-value |
| 1 Year | 2.6391 | 0.0263 | 100.53 | < 0.001 |
| 73 Years | 0.2326 | 0.0352 | 6.61 | < 0.001 |
| 473 Years | 0.1843 | 0.0313 | 5.90 | < 0.001 |
| **(B) Global effect of lava flow age** | | | | |
| Test | | χ² | df | P-value |
| Likelihood ratio test | | 19.77 | 2 | < 0.001 |
| **(C) Tukey-adjusted pairwise comparisons** | | | | |
| Comparison | | Estimate | z value | P value |
| 1 vs 73 years | | −0.2326 | −6.61 | < 0.001 |
| 1 vs 473 years | | −0.1843 | −5.90 | < 0.001 |
| 73 vs 473 years | | 0.0483 | 1.67 | 0.22 |
