# Supplementary material for: Benthic communities on recent and historical volcanic lava flows of an oceanic island
Source: PLoS One. 2026 Sep 23;21(9):e0357447. doi: 10.1371/journal.pone.0357447 (PMC13600511; doi:10.1371/journal.pone.0357447)
Supplement: Supplementary file 8 [file pone.0357447.s008.docx]

**Supplementary Table S8.** Results of the Conway–Maxwell–Poisson generalized linear model (COM-Poisson GLM) assessing differences in species richness of sessile and slow-moving benthic microinvertebrates associated with rocky substrates across lava flows of different ages (<1 year, 73 years, 473 years).

| (A) **Model coefficients (COM-Poisson GLM)** | | | | |
| --- | --- | --- | --- | --- |
| Term | Estimate | SE | Z value | P-value |
| 1 Year | 2.3026 | 0.0855 | 26.94 | < 0.001 |
| 73 Years | 0.5158 | 0.1077 | 4.79 | < 0.001 |
| 473 Years | −0.1054 | 0.1243 | −0.85 | 0.40 |
| **(B) Global effect of lava flow age** | | | | |
| Test | | χ² | df | P-value |
| Likelihood ratio test | | 17.51 | 2 | < 0.001 |
| **(C) Tukey-adjusted pairwise comparisons** | | | | |
| Comparison | | Estimate | z value | P value |
| 1 vs 73 years | | −0.516 | −4.79 | < 0.001 |
| 1 vs 473 years | | 0.105 | 0.85 | 0.67 |
| 73 vs 473 years | | 0.621 | 5.57 | < 0.001 |
